# The ownership memory self-reference effect shifts recognition criterion but not recognition sensitivity

**DOI:** 10.1007/s00426-024-01994-1

**Published:** 2024-06-21

**Authors:** S. D. Sparks, A. Kritikos

**Affiliations:** School of Psychology, The University of Queensland, St Lucia, Brisbane, 4072 Australia

**Keywords:** Ownership, Memory self-reference effect, Source memory, Sensitivity, Criterion

## Abstract

Information referenced to the self is retrieved more accurately than information referenced to others, known as the memory self-reference effect. It is unclear, however, whether social context (identity of the other) or task factors alter decision-making processes. In a virtual object allocation task, female participants sorted objects into their own or another’s (stranger or mother) basket based on a colour cue. Subsequently, they performed a recognition memory task in which they first indicated whether each object was old or new, and then whether it had been allocated to themselves or to the other. We obtained owner-specific hit rates and false-alarm rates and applied signal detection theory to derive separate recognition sensitivity (*d*’) and recognition criterion parameters (*c*) for self- and other-owned objects. While there was no clear evidence of a recognition self-reference effect, or a change in sensitivity, participants adopted a more conservative recognition criterion for self- compared with other-owned objects, and particularly when the other-referent was the participant’s mother compared with the stranger. Moreover, when discriminating whether the originally presented objects were self- or other-owned, participants were biased toward ascribing ownership to the ‘other’. We speculate that these findings reflect ownership-based changes in decisional processing during the recognition memory self-reference paradigm.

Our cognitive, perceptual and motor systems are sensitive to the self-relevance of objects and events in our environment (Cunningham & Turk, 2017; Humphreys & Sui, 2016; Kritikos et al., 2020; Sparks et al., 2016a). Information associated with the self, for example, own name (Bargh & Pratto, 1986; Moray, 1959; Yang et al., 2013) and own face (Sui & Han, 2007; Sui & Humphreys, 2013) has a processing advantage. The advantage is also seen at recall: typically, participants rate the extent to which trait adjectives describe either themselves or another person (Rogers et al., 1977), or whether they or another person ‘like’ an object (Ross et al., 2020), followed by a surprise recognition memory test (in which a judgement is made about whether a stimulus has been encountered previously) for the original words or objects plus new foils. Participants recognise and recall self-referenced words at a higher rate than other-referenced words, the memory *self-reference effect* (mSRE).

The mSRE is evident even for transient, virtual associations between the self and images of objects created during laboratory tasks where there is no explicit, self- or other-referential encoding (Cunningham et al., 2008; Cunningham & Turk, 2017; Klein, 2012; Sui et al., 2012; Turk et al., 2008; Zhu et al., 2007). In the ‘shopping task’ developed by Cunningham and colleagues (Cunningham et al., 2008), pairs of participants are informed that one basket (red or blue) belongs to them while the other belongs to the other person. They are asked to imagine they have ‘won’ the objects and sort a series of cards with the object images into the baskets, based on a red or blue colour cue. Thus objects are explicitly linked to self but self-knowledge is not required at encoding. In a subsequent surprise recognition memory test which includes the original plus new foils, participants indicate whether they have seen the object previously. The typical finding is enhanced recognition memory for self- compared with other-owned objects, the object mSRE (Cunningham et al., 2011; Englert & Wentura, 2016; Grisdale et al., 2014; Sparks et al., 2016a; Turk et al., 2013; Turk et al., 2011a, b; van den Bos et al., 2010).

In adaptations of the object mSRE task, both Turk et al. (2011b) and Kim and Johnson (2012) reported modulation of medial prefrontal cortex (mPFC) activity based on ownership status during the object allocation phase. In a later study, Kim and Johnson (2014) showed a similar modulation of mPFC activity when participants simply viewed self- compared with other-owned objects, even though neither the objects or ownership status were task-relevant, suggesting the mPFC is involved in incidental processing of object self-relevance (Cunningham & Turk, 2017; Humphreys & Sui, 2016; Northoff et al., 2006). Moreover, mPFC activation at object allocation is associated with memory performance for self-owned objects in the subsequent memory test (Turk et al., 2011b; Kim & Johnson, 2012). Thus it is possible that self- compared with other-owned objects receive a boost to attentional processing at encoding, and that this manifests in enhanced and/or more accessible recollective experiences of the encoding event for self-owned objects.

Recent studies show that self-prioritisation as well as the mSRE can be reduced or enhanced depending on behavioural and contextual factors such the identity of the other (Bentley et al., 2017; Clarkson et al., 2022; Sparks et al., 2016a) and the response modes imposed at the decision stage of the memory task (Conway & Dewhurst, 1995; Conway et al., 2001; Turk et al., 2013; van den Bos et al., 2010). These are important developments because they address the mechanisms of cognitive processing as well as the social context, reflecting real-world circumstances.

## Identity and the Relationship between Self and other

In preliminary studies, social context and the relationship of the other to the self modifies the self-prioritisation effect (SPE). Specifically, responses to information associated with ‘mother’ or ‘friend’ are faster than to ‘stranger’, although responses to self-relevant information are fastest overall (Sui et al., 2012, 2014). The magnitude of the mSRE may also depend on social context and the relationship between the self and the other. Bentley et al. (2017) investigated social in/exclusion and recognition of self- versus other-referenced trait words. While social exclusion was associated with the typical pattern of mSRE, inclusion abolished it such that participants recognised a comparable number of self- and other-referenced words (Bentley et al., 2017). Clarkson et al. (2022) modulated the object mSRE using a source memory paradigm. Source memory may be thought of as a more complex recollection process than recognition, because it requires binding multiple features of an event, including context (Craik et al., 1990; Raj & Bell, 2010). Clarkson et al. (2022) varied the amount of information made available about a virtual stranger other and showed that when participants received no information, there was clear source memory advantage for self- over other-referenced information, that is, for the context in which the information was encountered. When they were given information about the other (name, photo, hobbies and studies) and provided information about themselves, this advantage was abolished (Clarkson et al., 2022, 2024).

The above findings regarding the other in relation to the self are consistent with conceptualisations of expansion of the self to include significant others whose experiences, cognitions, memories and perspectives are bound with our own (Aron et al., 1991, 2004). The importance of the closeness of the relationship with the other is evident in the pattern of findings for self-prioritisation tasks, where shape-name pairings are categorised fastest for the self, but faster for close others (mother, friend) than stranger (Humphreys & Sui, 2016; Sui & Humphreys, 2013; Sui et al., 2012). Accordingly, the mSRE magnitude is attenuated when the other is significant (Clarkson et al., 2022), such as mother compared with a stranger, with further attenuation for Chinese individuals (Golubickis et al., 2019; Sparks et al., 2016a). This may be because of overlapping cognitive representations for self and close-other information, for example, in verbal semantic categories (Mashek et al., 2003) as well as facial feature representations (Ketay et al., 2019). Sparks et al. (2016a) speculated that the attenuation of the mSRE for the mother condition in Chinese individuals may have been attributable to differences in inter-dependent construals between the Chinese and Australian participants (Sparks et al., 2016a; Kitayama & Uskul, 2011; Markus & Kitayama, 2010). It possible, therefore, that a reduction or abolition of the mSRE may be observed in conditions where the other represents a close social relationship (such as mother), compared with the more typical stranger other.

## Cognitive decisions at the Retrieval Stage

Modulations in recognition memory performance indicate underlying decision-making differences in linking information to the self or the other. Typical hit rate analysis for recognition accuracy may not capture the extent and range of these differences. In evidence accumulation models, it is postulated that evidence for decision alternatives accumulates at a certain rate, until a threshold is reached and a decision is triggered. For example, Golubickis and colleagues (2018, 2019) investigated the rate of evidence accumulation with hierarchical drift diffusion models, and showed pre-decisional bias towards responding ‘self’ in self- and other-ownership tasks. Moreover, response requirements, such as two- versus three- alternate forced choice options, generate variations in the decision processes and hence memory performance (Schurgin, 2018).

The original object mSRE paradigms used a yes/no judgement at retrieval where participants respond to whether they saw the original or foil object previously, thus calculating recognition memory accuracy based on hits and false alarms (Cunningham et al., 2008). Using this ‘one-step’ response, self-owned objects are more likely than other-owned ones to evoke specific, recollective experiences of viewing the object during the object allocation task (Turk et al., 2013; van den Bos et al., 2010). This ‘one-step’ response, however, allows only an overall calculation of recognised self- and other-referenced objects. It does not disambiguate between recognition of objects *specifically* attributed to the self versus the other, sensitivity or shift in criterion, or the confidence in the decision. Moreover, when using ‘one-step’ paradigms, comparisons of hit rates do not reveal nuanced underlying biases in decision-making, such as sensitivity or bias. To address this, one study (Vella et al., in preparation) investigated recognition memory responses in the object mSRE with a hierarchical Linear Ballistic Accumulator model, and found shifts in criterion towards responding ‘self’, rather than the rate of information accumulation, was the dominant mechanism responsible for alterations in performance. Thus, many mechanisms that underpin the nature of the decision at the retrieval stage of the object mSRE are unclear.

These limitations have been addressed with adjustments to the response requirements. Within memory paradigms generally, recollection (‘remember’) responses are considered to represent specific details of an event. In contrast, familiarity (‘know’) responses reflect memory strength but not specific details, and can be thought of as a signal detection process of discriminating between old vs. new items. The two instructions (old/new vs. remember/know), yield performance differences (Schurgin, 2018; Yonelinas et al., 2010). van den Bos et al. (2010) added a remember/know/guess judgement (Tulving, 1985) to word-based SRE tasks (Conway & Dewhurst, 1995; Conway et al., 2001). In a two-step recognition memory test with a remember/know/guess choice, participants provide a ‘recognise’ response during the memory test, and subsequently indicate whether they consciously recollect the object from the encoding task (‘remember’), have no specific recollection of the object but have a feeling of ‘knowing’ that it has been in the encoding task (‘know’), or merely guess the object is old without any recollection of it or feeling of knowing. Self-referenced words evoked a higher proportion of ‘remember’ relative to ‘know’ responses than did other-referenced words (Conway & Dewhurst, 1995; Conway et al., 2001). van den Bos et al. (2010; see also Turk et al., 2013; Vella et al., in preparation) used the same response choice with the object mSRE paradigm, and showed that self-owned objects prompted more ‘remember’ responses compared with other-owned ones, whereas the number of ‘know’ responses did not differ according to ownership (van den Bos et al., 2010). In other words, the object mSRE emerged for ‘remember’ but not ‘know’ responses, suggesting that encoding an object in the context of self- compared with other-ownership increases the likelihood that viewing the object later will evoke specific, episodic recollections of the encoding event, and hence improves memory performance.

It is also possible that decisional biases based on the *content* of the memory traces during the memory test contribute to higher hit rates for self- compared with other-owned objects. In a recognition memory test, participants must decide whether or not their memory-based recall of each presented object warrants a ‘recognise’ response. The encoding context of the objects may bias this decision. For example, if self- rather than other-owned or new objects in real-life (e.g., pair of trainers) are more likely to evoke a sense of ‘mine-ness’ during the memory test, participants may have a bias toward responding ‘recognise’ rather than ‘do not recognise’. Such a bias would inflate the hit rate for self- relative to other-owned objects, and there may be an interaction with the likelihood of owning it (Mueller et al., 1988). Similarly, if the task includes a remember/know judgement, a higher rate of ‘remember’ hits will emerge for self- compared with other-owned objects if participants are biased toward selecting ‘remember’ responses for objects that evoke a sense of self-ownership.

Most studies using the Cunningham et al. (2008) memory object mSRE paradigm rely on recognition memory measures that do not isolate decisional bias (Sparks et al., 2016a; Turk et al., 2011a, b, 2013; van den Bos et al., 2010). Participants give ‘old’ and ‘new’ responses to previously presented and new (foil) objects respectively, generating unique hit rates for self- and other-owned objects, but a common false alarm rate which is then used to calculate Corrected Hit Rates (CHRs; i.e., hit rate minus false alarm rate), the measure of recognition performance. Because of this common false alarm rate, any difference in CHR is identical to the difference that existed in the uncorrected hit rates. Correcting hit rates using the common false alarm rate is useful to establish that participants’ recognition performance is above chance level, but does not correct for potential differences in decisional bias between self- and other-owned objects. For this reason, differences in CHRs for self- compared with other-owned objects could reflect differences in the strength and/or accessibility of memory traces, in decision bias based on the content of these memory traces, or a combination of these factors. Some studies (Clarkson et al., 2022; Cunningham et al., 2013, 2014; Pereira et al., 2021) have taken account of these differences in response mode and the refined information it generates about recall.

A further consideration is the quality of the memory trace during decision judgements, investigated though the sensitivity (quality of the memory match signal between the object displayed and the retrieved memory trace) and criterion (decision bias, or threshold the memory match signal must meet for the participant to respond ‘recognise’ rather than ‘do not recognise’) calculations proposed by signal detection theory (Banks, 1970; Lockhart & Murdock, 1970; Schurgin, 2018; Shapiro, 2014). In general word memory recognition tasks some participants are systematically liberal or conservative, that is, tend to respond that a stimulus has or has not been encountered (Kantner and Londsay, 2010; 2012). In these paradigms, they tend to respond ‘recognise’ when the memory match signal is relatively strong versus relatively weak. Important for the object mSRE, participants could differentially adopt a more liberal criterion for objects that evoke a sense of self-association compared with objects that do not. Calculating sensitivity and criterion for a given condition requires both a hit rate and false alarm rate distinctly for self- and other-owned objects.

Some object mSRE studies have used a source-specific recognition task where participants responded ‘Me,’ ‘Other’, or ‘New’ during the memory test (Cunningham et al., 2011, Experiment 3; Cunningham et al., 2013; Cunningham et al., 2014; Pereira et al., 2021). Cunningham et al. (2011) calculated sensitivity (d’) based on unique self-owned and other-owned false alarm rates. Prior to the allocation tasks, participants were able to self-select numbers corresponding to unidentified objects, but were also allocated objects ostensibly selected by the other - although in fact all objects were randomly allocated. For the ‘self-chosen’ objects, sensitivity was higher for self- than other-owned objects, but this difference was only marginally significant. For ‘other-selected’ objects, ownership did not affect sensitivity, suggesting ownership alters sensitivity when participants believe they have chosen the self-owned objects. In this paradigm, however, participants selected numbers associated with objects. Thus it is unclear if the changes in sensitivity generalise to the standard paradigm in which participants do not select the objects they are allocated. A prominent gap in the literature is that subtle aspects of responses have not been interrogated. Measures such as sensitivity and criterion may provide a refined understanding of subtle memory and decision-making processes in the object mSRE, beyond hit rates. A second prominent gap revolves around the identity of the other. In preliminary work we have shown that the identity of the other can modulate the mSRE (Clarkson et al., 2022; Sparks et al., 2016a), but the impact of social relationships on sensitivity and criterion in self- and other-referenced recall is unknown. Specifically, if recall is attenuated for a close other (such as mother) compared with stranger, it is possible that there will also be dissociations in recollection and familiarity, and hence sensitivity and criterion, for the two groups.

## The Present Study

In this experiment, we investigated social context and decisional factors that modulate the object mSRE in a two-step response memory retrieval task. Specifically, we analysed responses in terms of common and specific false alarm rates to enable separate calculations of recognition (old vs. new item) and source (self vs. other items) memory calculations respectively, as well as distinct calculations of sensitivity and criterion shifts for recognition and source memory. Participants sorted consecutively presented objects into their own or another’s (stranger, mother) bag based on red or blue colour cues. Subsequently, the original objects plus foils were again presented. Participants were initially asked if they recognised each object in turn; if they responded ‘yes’, they were asked whether it had been allocated to themselves or to the other. In this way, we were able to calculate distinct hits and false alarms for self- vs. other-referenced objects that are recognised, as well as separate sensitivity and criterion scores.

To account for potential differences in performance, we also manipulated the identity of the other: for one group, the other was a female stranger, while for the second group the other was their mother. Because females are generally considered to show interdependent self-construals (Cross & Madson, 1997), we recruited and tested only female participants from a Western background to reduce variability in decision-making, and to ensure gender congruence for the self-other relationship. Moreover, because we investigate close-other relationships, we obtained self-ratings of Closeness and Similarity to the other to correlate with recognition and source memory performance.

Overall, we expected the mSRE to be evident in both recognition memory, such that, indexed by higher CHRs, objects allocated to the self should be recalled more accurately overall. Moreover, participants should identify items as having been allocated to themselves more accurately than objects allocated to the stranger or mother (source memory). For the self-stranger group, we also expected to see an object mSRE, such that recognition and source memory accuracy are higher for self- than for stranger-referenced objects. By comparison, for the self-mother group, because they are Western participants, we expected an object mSRE such that recognition and source memory accuracy should be higher for self- than for mother-referenced objects. While we expected a recognition and source memory object mSRE for both groups (because participants were from Western background) we expected it would be reduced in magnitude for the self-mother group, that is, there should be an interaction between object owner and other identity.

We also expected that participants would show higher sensitivity (d’) as well as a more liberal criterion (c) for old, self- compared with other-owned objects, regardless of the identity of the other (stranger or mother). Criterion difference may be amplified for the self-mother group. These differences are likely to be seen more clearly for the source-specific analyses, when correcting for self- versus other-specific false alarms, than in the uncorrected false alarm analyses using false alarms common to self- and other-owned objects, as in recognition memory calculations.

Finally, we expected that self-reported ratings of Closeness and Similarly would be correlated with mSRE, such that increased Closeness and Similarity would be associated with reduced recognition memory and reduced source memory for self- compared with other-referenced objects.

## Method

### Participants

One-hundred-forty-five female students from a Western culture enrolled in first-year psychology courses at the University of Queensland voluntarily participated in exchange for course credit. Ethics approval was obtained from The University of Queensland Ethics Committee. (We expand upon the decision to recruit Western, female participants in the Generalisability section of the Discussion.) One participant withdrew early in the session, and four participants’ data were lost due to unscheduled downtime on data servers. The remaining participants all self-identified as White/European/Caucasian with English as a first language, and their mean age was 20.76 years (range 17 to 53, SEM = 0.55, SD = 6.50, median = 18).

### Stimuli & Procedure

Between one and seven participants completed the experiment in-person during each session, sitting within individual cubicles. The LCD computer monitors (Dell P2414Hb, 60.47 cm diagonal, 52.70 cm width, 29.60 cm height) were set to 1920 × 1080 resolution and 60 Hz refresh rate throughout the session. Participants viewed the screen from a distance of approximately 60 cm, although this varied according to participants’ height and posture. As per previous studies, head movements were not constrained.

#### Introductory phase

The introductory phase was presented via Qualtrics in a web browser. The first page introduced a woman named ‘Emily’ in the Stranger condition or instructed participants to think about their mother for the Mother condition.

##### Stranger introduction

Participants in the Stranger condition viewed a photo of ‘Emily’, a Caucasian woman in her early 20s (participants were not provided her age). The onscreen text instructed them to read a brief description of Emily carefully, which was as follows: *“Hello, my name is Emily! My hobbies are reading, baking and going out with my family. I enjoy watching dramas. My favourite kinds of music are classical music and indie pop.”*

The following prompts requested participants to list their favourite hobby or hobbies, their favourite television show(s) or film(s), and their favourite type(s) of music.

##### Mother introduction

Participants in the Mother condition were prompted to think about their mother, including her appearance and her hobbies and interests. The following prompts requested they list first their mother’s then their own favourite hobby or hobbies, their favourite television show(s) or film(s), and their favourite type(s) of music.

##### Demographic information

After the Other Identity manipulation, the next page prompted participants to provide their age, handedness, gender, and whether or not they were colourblind (yes, no, unsure), their general cultural background (Western/Caucasian or Asian), further free-response information about their cultural background, and the cultural background of their parents. Next they were prompted to provide the time they had lived in Australia, list the countries they had lived in, and identify what they considered their home country. We used data from these questions to identify participants who met the eligibility criteria for the current study.

#### Object allocation (encoding) task and surprise memory test

The experimental phase consisted of an object allocation encoding task and a surprise memory test which drew on a colour image set of 150 household objects previously used by Sparks et al. (2016a) and van den Bos et al. (van den Bos et al., 2010), and are part of a larger set of objects used in numerous previous studies (Cunningham et al., 2008, 2011; Turk et al., 2013; Turk et al., 2011a, b). All of these components were presented via E-Prime 2.0 (Psychology Software Tools, Pittsburgh, PA; runtime version 2.0.10.356).

We divided the objects into three sets of 50 objects matched for approximate value and type (e.g., vegetable, appliance, clothing). The images ranged from 57 to 250 pixels wide and 91 to 250 pixels high. Randomly for each participant, one set was allocated to be self-owned, one other-owned, and one to be the new/foil objects in the surprise memory test.

##### Encoding Task

After being prompted to think about the other (Emily or Mother), participants received onscreen instructions for the encoding task, described as a ‘shopping task’. One red and one blue shopping bag appeared on either side of the screen. Participants were instructed that one belonged to them and the other belonged to the other (Emily or Mother). The side and colour of bag allocated to the participant and the Other was randomised. Participants were instructed that a series of everyday objects would appear in the centre of the screen, shortly followed by red or blue colour bars above and below it. They were instructed to press and hold the left and right arrow keys to move each object into the appropriate bag according to the colour cues. They were told that everything going into their bag belonged to them and everything going into the other bag belonged to the other. Participants were then prompted to press the arrow key that corresponded to their bag and then to the other’s bag to confirm they had understood who owned each bag and its contents.

Each trial of the encoding task began with the two bags positioned to the left and right side of a white background, anchored 600 pixels away from the centre of the display. After 500ms, the object appeared in the centre of the screen. 1500ms later, the red or blue bars appeared above or below the object. Participants had up to 2000ms to initiate movement of the object, then a further 5000ms to move the object completely into the bag. A smooth movement from the starting position of the object into the bag took 1670ms (exact timing depended on when movement initiation occurred relative to the refresh cycle of the monitor). Once participants had moved the object into the bag or the trial had timed out, the red or blue bars disappeared (as well as the object in the case of a time-out) and the next trial began. The order of objects was randomised.

At the end of the allocation task we administered a filler task (mean duration 6.50 min median = 6.33, SD = 1.24, range 4.24 to 15.77), which was a computerised adaptation of the Kitayama et al. (2003) Framed Line Test. Participants viewed a target stimulus consisting of a square box with a vertical line extending downwards from the centre of its top side. After an intervening flash of visual noise, participants viewed a different-sized square and adjusted the relative or the absolute length of a vertical line within the second square.

##### Surprise Memory Test

Participants were informed that they would view a series of objects and asked to indicate whether they recognised them from the ‘shopping task’ or whether they were new. Objects were presented centrally in random order and initially appeared with the ‘Recognise?’ prompt at the bottom centre of the screen. From the onset of each object, participants had up to 5000ms to indicate whether they recognised (O key) or did not recognise (P key) the object. If participants indicated they recognised the object, the text prompt changed to ‘Whose was it?’ and they had up to 5000ms to indicate if the object belonged to them (Q key) or the Other (A key). If they indicated they did not recognise the object or failed to respond within the allotted time the trial ended. There was a 1000ms intertrial interval consisting of an empty white screen.

As manipulation checks, subsequent to the memory task, participants were asked to recall who owned the red and blue bags. For the Stranger condition, they completed multiple choice questions testing their memory of Emily’s hobbies and interests. For the Mother condition only, participants rated the current frequency of their contact with their mother and whether they had been in regular contact with their mother during childhood.

### Closeness and similarity ratings

Participants completed closeness and similarity ratings with respect to the other referent (Stranger/Emily or Mother), by positioning a marker on a line anchored with “You” (i.e., the participant) on the left and Emily or Mother on the right. These data were coded from 0 (least close to self) to 100 (closest possible to self). For similarity ratings, participants positioned a marker on a line with “just like me” (left), “neither like me or not like me” (centre), and “not at all like me” 96 (right) as the anchors. The same set of referents was used as in closeness ratings. The program scored ratings from 50 (maximum similarity to self) to -50 (maximum dissimilarity to self), with 0 as the neutral point.

## Results

### Calculation of memory parameters

#### General CHR (CHR; Recognition Memory)

General CHR was calculated separately for self- and other-owned objects using a shared false alarm rate which is the proportion of ‘recognise’ responses to new (foil) objects (see Cunningham et al., 2008; Sparks et al., 2016a; Turk et al., 2013).

Self-owned hit rate was the proportion of ‘recognise’ responses to self-owned objects and other-owned hit rate was the proportion of ‘recognise’ responses to other-owned objects. General CHR for self-owned objects was the self-owned hit rate minus the shared false alarm rate. General CHR for other-owned objects was the other-owned hit rate minus the shared false alarm rate. For ease of reading, we converted general CHRs from the proportion format (0.00 to 1.00) to the percent format (0 to 100).

#### Source-specific CHR (source memory)

Similar to Cunningham et al. (2011), we calculated source-specific CHRs for self- and other-owned objects using source-specific false alarm rates. For self-owned objects, the source-specific hit rate was the proportion of self-owned objects that each participant correctly recognised *and* correctly attributed to the self. The source-specific false alarm rate for self-owned objects was the proportion of new objects to which each participant incorrectly responded ‘recognise’ *and* subsequently attributed to the self. Source-specific CHR for other-owned objects was calculated in the equivalent manner. For ease of reading, we converted source-specific CHRs from the proportion format (0.00 to 1.00) to the percent format (0 to 100).

#### Signal Detection Parameters

We calculated signal detection parameters using a loglinear approach for dealing with false alarm and hit rates equal to 0 or 1 without biasing the resulting parameters (see Stanislaw & Todorov, 1999, p 144). For all participants, this approach consists of adding 0.5 to the number of hits and the number of false alarms and adding 1 to the total number of signal and noise trials before calculating any signal detection parameters.

Sensitivity (*d*’) was calculated as *d*’ = F^− 1^(H) - F^− 1^(F), where H is the hit rate and F is the false alarm rate. A d’ of 0.0 corresponds to chance performance, and the more positive the *d*’ value, the higher the sensitivity.

Criterion (c) was calculated as c = -(F^− 1^(H) + F^− 1^(F))/2, where H is the hit rate and F is the false alarm rate. A c of 0.0 represents an unbiased criterion. Increasingly positive c values indicate a correspondingly positive conservative criterion, that is, the participant requires a stronger signal before they respond that the target is present, and are *less likely* to indicate the target is present when uncertain. Conversely, negative c values indicate a more liberal criterion, that is, the participant tends to respond that the target is present even for weaker signals, and are *more likely* to indicate the target is present when uncertain.

#### Discriminating self- and other-owned objects from New objects

The primary signal detection analysis was based on the sensitivity and criteria for discriminating self-owned from new (foil) objects and for discriminating other-owned objects from new (foil) objects. For self-owned objects, a hit was defined as correctly responding ‘recognise’ to a self-owned object and correctly attributing it to the self. A false alarm for self-owned objects was defined as incorrectly responding ‘recognise’ to a new (foil) object and incorrectly attributing it to the self. Hits and false alarms for other-owned objects were defined equivalently. We will refer to these parameters with the labels *self-versus-new d’, self-versus-new c, other-versus-new d’*, and *other-versus-new c.*

#### Discriminating self-owned objects from other-owned objects

The secondary signal detection analysis was based on sensitivity and criteria for discriminating self-owned from other-owned objects. For this analysis, we arbitrarily considered self-owned objects the ‘signal’ and other-owned objects the ‘noise’. Thus, a hit was defined as responding ‘recognise’ to a self-owned object and correctly attributing it to the self. A false alarm was defined as correctly responding ‘recognise’ to an other-owned object but incorrectly attributing it to the self. We will refer to these parameters with the labels *self-versus-other d’* and *self-versus-other c.*

### Data screening

To ensure that participants who did not engage with or attend to the task did not bias the data or introduce noise to the analysis, they were included in the group-level analyses if they passed the following checks: correctly completing at least 95 out of 100 trials of the object allocation task (i.e., object moved into the cued bag within the maximum allotted time); correctly identifying their own and the other’s bag colours both immediately before the memory test and at the end of the questionnaire phase; responded in time to at least 145 out of 150 of the ‘Recognise?’ prompts; overall CHR in memory test above 0 (chance level); overall sensitivity (d’) in memory test above 0 (chance level).

Of the original 140 participants, 126 remained (67 stranger condition, 59 mother condition; *M*_age_ = 20.39 years SD = 5.65), achieving high statistical power (> 95%) to detect a medium-sized ownership effect (d_z_ = 0.50) comparable with Cunningham et al. (2008), and acceptable statistical power (80%) to detect a small-to-medium ownership effect (d_z_ ≧ 0.25), as calculated with G*Power 3.1.9.4, (Faul et al., 2007).

### General CHR analysis – recognition memory

**Fig. 1:**
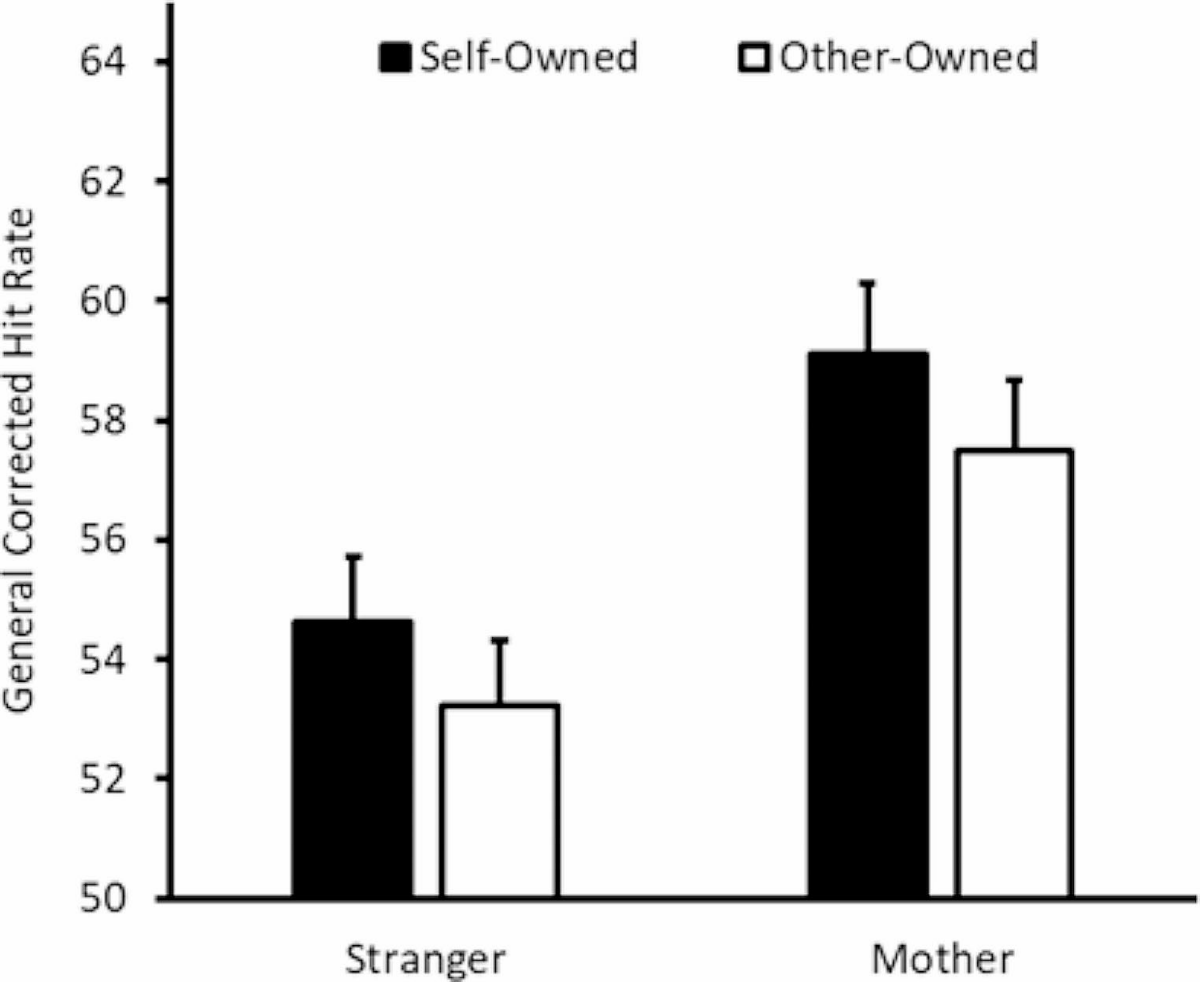
General CHR as a function of object owner (black versus white fill) and other type (X axis). Error bars represent one standard error of the difference for the ownership comparison within each group

To investigate our hypothesis that the recognition memory would be higher for self-referenced objects, we submitted participants’ general CHRs to a mixed 2 × 2 ANOVA with object owner (self vs. other) as a within-subjects factor and other identity (stranger vs. mother) as a between-subjects factor. There was a trend towards significance for the object owner main effect (*F*(1, 124) = 3.55, *p* = .062, h_p_^2^ = 0.03, MSE = 40.56), such that hit rates were higher for self- than other-owned objects (*M* = 56.87, *SEM* = 1.75 and *M* = 55.36, *SEM* = 1.67 respectively). The other identity main effect (*F*(1,124) = 1.73, *p* = .191, η_p_^2^ = 0.01, *MSE* = 695.44) and object owner by other identity interaction (*F*(1,124) = 0.02, *p* = .889, h_p_^2^ < 0.01, *MSE* = 40.56) were nonsignificant. The general CHR data are displayed in Fig. 1.

### Source-specific CHR – source memory

**Fig. 2:**
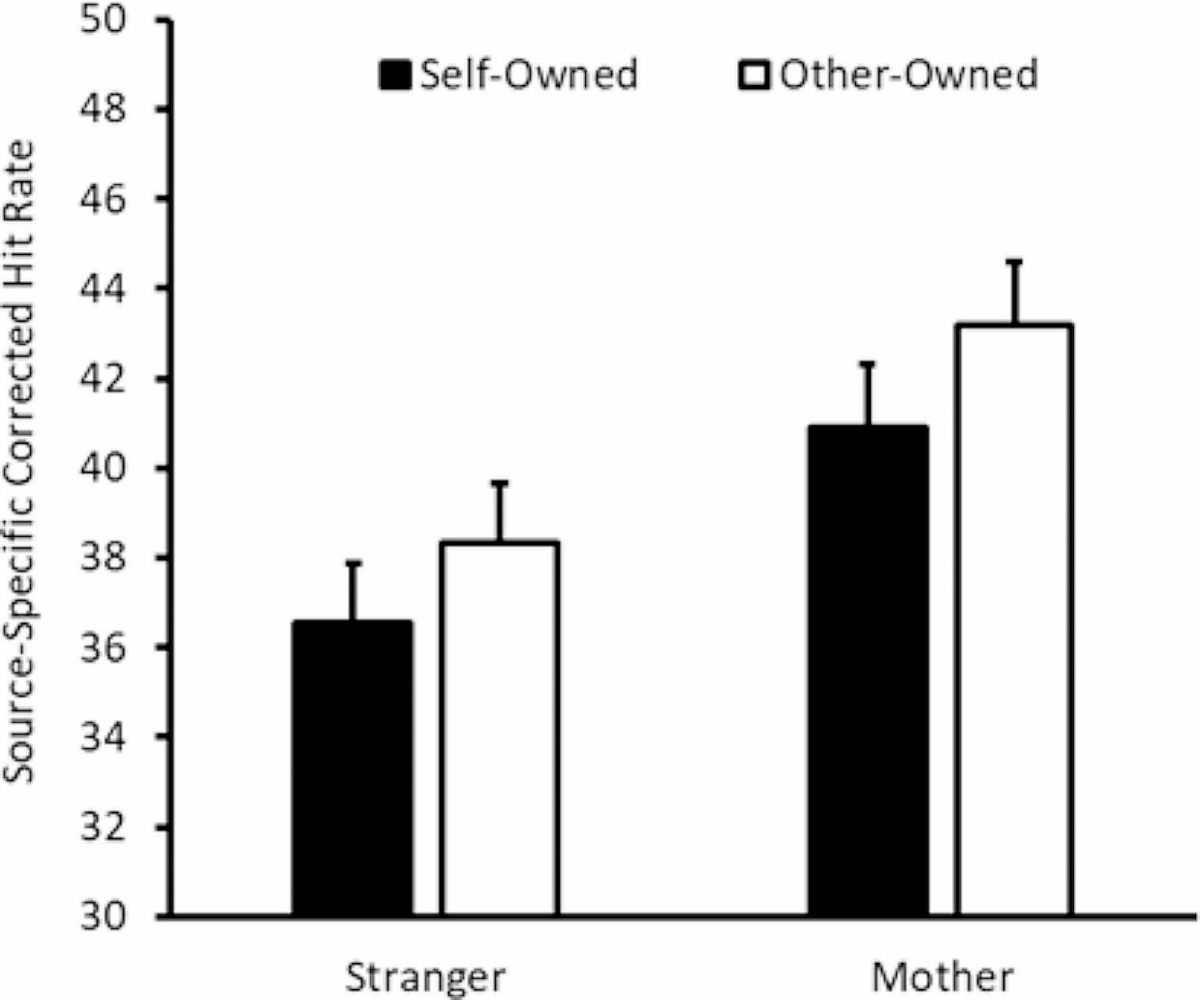
Source-specific CHR as a function of object owner (black versus white fill) and other type (X axis). Error bars represent one standard error of the difference for the ownership comparison within each group

To investigate our hypothesis that the source memory would be higher for self- than other-referenced objects, we submitted participants’ source-specific CHRs to a mixed 2 × 2 ANOVA with object owner (self vs. other) as a within-subjects factor and other identity (stranger vs. mother) as a between-subjects factor. The object owner main effect was significant (*F*(1,124) = 4.35, *p* = .039, η_p_^2^ = 0.03, *MSE* = 59.50); unexpectedly, however, performance was lower for self-owned objects than other-owned objects *(M* = 38.73, *SEM* = 1.50 and *M* = 40.76, *SEM* = 1.63 respectively). The other identity main effect (*F*(1,124) = 2.40, *p* = .124, η_p_^2^ = 0.02, *MSE* = 556.92) and the two-way interaction (*F*(1,124) = 0.61, *p* = .806, η_p_^2^ < 0.01, *MSE* = 59.50) were nonsignificant. These data are plotted in Fig. 2.

### Sensitivity (d’) for discriminating self- and other-owned objects from New objects

Our next stated hypotheses referred to sensitivity and criterion (section below) for self- and other-allocated objects compared with new foils. We submitted participants’ d’ scores for owned objects versus new objects to a mixed 2 × 2 ANOVA with object owner (self vs. other) as a within-subjects factor and other identity (stranger vs. mother) as a between-subjects factor. The object owner main effect was not significant (*F*(1,124) = 1.74, *p* = .190, η_p_^2^ = 0.01, *MSE* = 0.19). The other identity main effect (*F*(1,124) = 2.30, *p* = .132, η_p_^2^ = 0.02, *MSE* = 1.76) and the two-way interaction (*F*(1,124) = 1.77, *p* = .186, η_p_^2^ < 0.01, *MSE* = 0.19) were nonsignificant.

### Criterion (c) for discriminating self- and other-owned objects from New objects

**Fig. 3:**
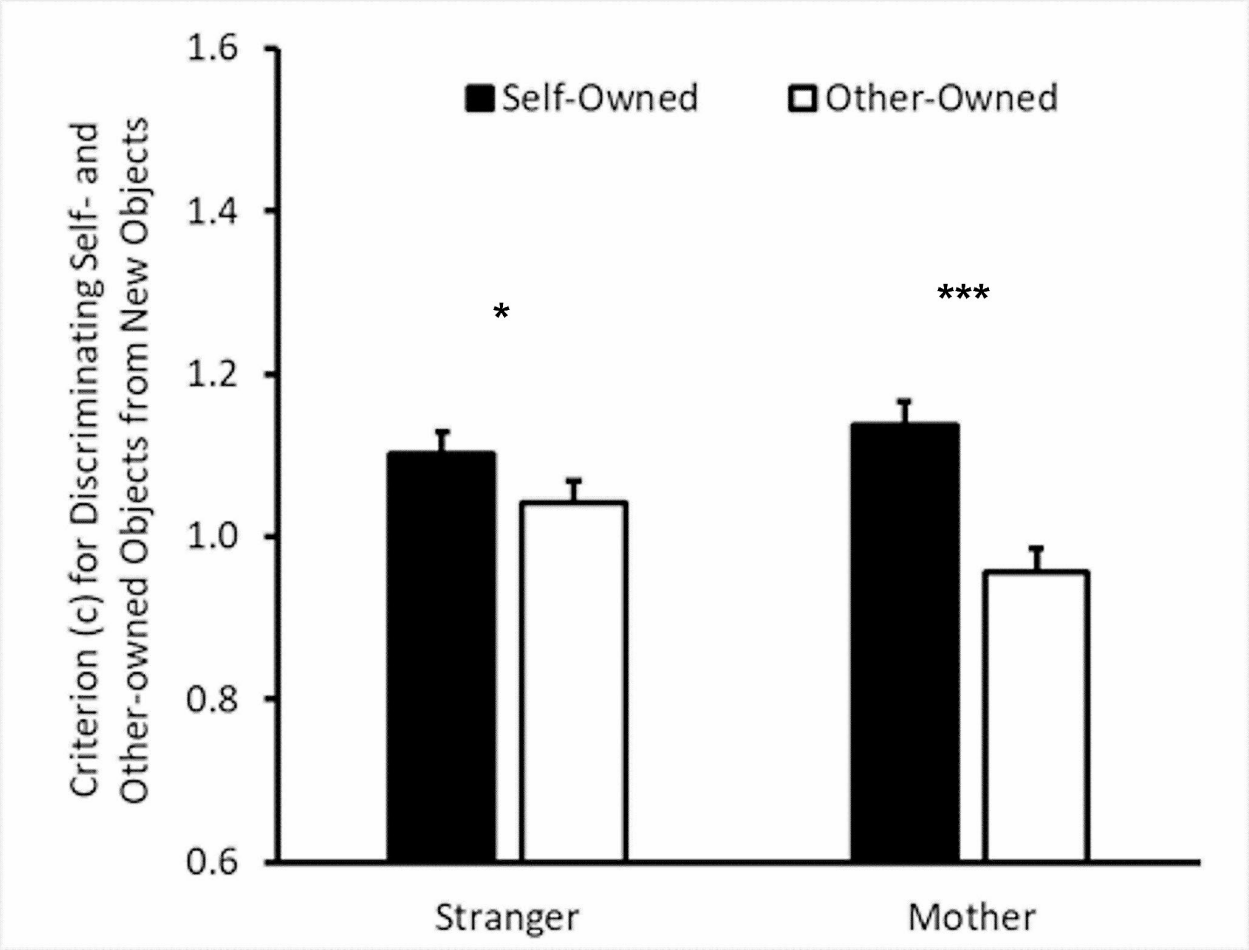
Criterion or discriminating self- and other-owned objects from new objects as a function of object owner (black versus white fill) and other type (X axis). Error bars represent one standard error of the difference for the ownership comparison within each group. *p = .02, ***p < .001

We submitted participants’ c scores for owned objects versus new objects to a mixed 2 × 2 ANOVA with object owner (self vs. other) as a within-subjects factor and other identity (stranger vs. mother) as a between-subjects factor. The main effect of other type was nonsignificant (*F*(1,124) = 0.28, *p* = .600, η_p_^2^ < 0.01, *MSE* = 0.14). The object owner (*F*(1, 124) = 36.80, *p* < .001, η_p_^2^ = 0.23, *MSE* = 0.03) main effect was significant, but this was qualified by a significant object owner by other identity interaction (*F*(1,124) = 9.02, *p* = .003, η_p_^2^ = 0.07, *MSE* = 0.03). Follow-up t-tests (Holm-Bonferroni corrected) were conducted on object owner effects within each other identity condition. For the stranger condition, participants showed a significantly more conservative self-versus-new criterion compared with their other-versus-new criterion (*M* = 1.10, *SEM* = 0.03 and *M* = 1.04, *SEM* = 0.04 respectively; *t*(66) = 2.38, *p* = .020, *SEM* = 0.03). In the mother condition, self-versus-new criterion was also significantly more conservative than other-versus-new criterion (*M* = 1.14, *SEM* = 0.03 and *M* = 0.96, *SEM* = 0.04 respectively; *t*(58) = 5.86, *p* < .001). The interaction appeared to be driven by a substantially smaller effect size for the stranger condition (d_z_ = 0.29) compared with the mother condition (d_z_ = 0.77). The data for criterion are graphed in Fig. 3.

### Sensitivity (d’) for discriminating self-owned from other owned objects

Turning to our hypotheses regarding sensitivity and criterion (section below) for source memory (self- vs other-allocated objects), a single-sample t-test revealed that self-versus-other sensitivity (*M* = 1.05, *SEM* = .07) was significantly above the chance level of d’ = 0.00, *t*(125) = 15.67, *p* < .001. A between-groups t-test showed no significant difference in self-versus-other d’ in the stranger group compared with the mother group (*M* = 0.99, *SEM* = 0.09 and *M* = 1.11, *SEM* = 0.10 respectively, *t*(124) = 0.92, *p* = .360.

### Criterion (c) for discriminating self-owned from other-owned objects

A single-sample t-test revealed that self-versus-other criterion (*M* = 0.15, *SEM* = 0.02) was significantly biased toward responding ‘other’ rather than ‘self’ compared with an unbiased criterion of *c* = 0.00, *t*(125) = 6.73, *p* < .001. A between-groups t-test showed no significant difference in self-versus-other criterion in the stranger group compared with the mother group (*M* = 0.13, *SEM* = 0.03 and *M* = 0.17, *SEM* = 0.03 respectively), *t*(124) = 0.86, *p* = .389.

### Correlations with closeness and similarity ratings

To investigate our hypothesis regarding recognition and source memory, and Closeness and Similarity to the other, we correlated these ratings with measures of self-bias. Two participants in the stranger condition did not provide ratings for Closeness and Similarity, thus *N* = 48. Participants rated themselves as significantly closer and more similar to the other in the mother compared with the stranger condition (*t*(89) = -17.432 *p* < .0001 and *t*(89) = -6.403 *p* < .0001; see Table 3. These two ratings did not correlate significantly with the self bias measures, for recognition memory or source memory (see Table 1).

**Table 1:** Means and standard deviations for the Closeness and Similarity ratings, and two-tailed non-parametric correlations (Spearman’s ρ, 2-tailed) with self-bias (self minus other) Recognition and Source self-bias, sensitivity (d’) and criterion (c) for the stranger (Emily) and mother groups

		Mean (SD)	Recognition CHR self-bias	Source self-bias	Self-bias d’	Self-bias c
Emily (Stranger) *N* = 48	Closeness	24.15 (22.57)	0.114 *p* = .439	0.168 *p* = .225	0.252 *p* = .084	0.037 *p* = .803
	Similarity	5.39 (18.55)	− 0.128 *p* = .386	− 0.176 *p* = .232	0.079 *p* = .593	0.174 *p* = .238
Mother *N* = 43	Closeness	91.35 (12.01)	− 0.273 *p* = .076	− 0.092 *p* = .556	0.253 *p* = .101	**0.357** ***p*** **= .019**
	Similarity	29.35 (16.69)	− 0.095 *p* = .543	0.117 *p* = .456	0.195 *p* = .209	**0.444** ***p*** **= .003**

## Discussion

In this paper we aimed to investigate decision-making processes in the recognition memory of self- and other-related information, in response to task-related factors such as response choices and the relationship between the self and the other. We obtained owner-specific hit and false-alarm rates and applied signal detection theory to derive separate recognition sensitivity (*d*’) and recognition criterion parameters (*c*) for self-owned and other-owned objects. We also derived sensitivity and criterion parameters for distinguishing self-owned from other-owned objects to test the extent to which participants could differentiate self-owned and other-owned objects. Female participants completed a computerised version of the Cunningham et al. (2008) ‘shopping task’ in which they sorted virtual objects into shopping bags belonging to the self and a female virtual ‘other’ who was either a stranger or their mother. Subsequently, participants completed a surprise memory task in which ‘old’ and ‘new’ foil objects were randomly and sequentially presented. In a two-step response choice mode, participants indicated whether they did or did not recognise the object. If they responded that they recognised it, they were the prompted to indicate whether it belonged to themselves or the ‘other.’ Thus, self-specific false alarms were differentiated from other-specific false alarms.

### Summary of results

Overall, we found clear evidence that ownership affected recognition criterion. Participants adopted a more conservative recognition criterion for self- compared with other-owned objects. Interestingly, this effect was stronger for mother compared with stranger (Emily). Furthermore, when discriminating whether the originally presented objects were self- or other-owned, participants were biased toward ascribing ownership to the other, suggesting that ownership-related decisional biases are important in the object mSRE paradigm. In contrast, we found no evidence that ownership affected recognition sensitivity. Thus, our data do not support the notion that self-owned objects are easier to distinguish from new objects than are other-owned objects. Participants were able to discriminate reliably self- from other-owned objects, however.

Finally, to facilitate comparison to previous studies, we analysed CHR data using a shared false alarm rate and using owner-specific false alarm rates (recognition and source memory respectively). Interestingly, no ownership effects emerged when hit rates were corrected with a shared false alarm rate, and a *reversed* ownership effect (self-owned < other-owned) occurred when owner-specific false alarm rates were used. These CHR findings were unexpected, and we speculate that there are attributable to the information about “Emily” given to the participant in the self-stranger group. This may have been sufficient to trigger a transient ‘relationship’ between the virtual stranger and the participant, by increasing the salience of the stranger and thus attenuating the mSRE, as shown in Clarkson et al. (2022). Related to this, and as a discussed below, with increased knowledge about the other, the decisional bias may have shifted such that participants had a more conservative criterion for indicating that an object was associated with themselves rather the other – that is, they were more willing to indicate the other (stranger or mother) was the owner of an object.

### Decisional Bias as a context-sensitive contributor to the object mSRE

Previous work in recognition memory has shown systematic shifts in bias depending on modality (pictures, words), that can be consistent within participants (Kantner & Lindsay, 2010, 2012). Here, our finding that recognition criterion differed as a function of ownership demonstrates that decisional biases also affect performance on the memory component of the task. Both sensitivity and criterion influence CHR, which is the typical measure of recognition performance. Thus it is possible that decisional biases that are sensitive to ownership in the *content* of the memory traces at least partially drive the typical object mSRE pattern of higher self- than other-CHRs found in previous research. This explanation requires that participants can access some form of ownership information during the memory test. In the current study, participants could discriminate reliably self- from other-owned objects during the memory test, which would only be possible if accessing ownership-relevant information is associated with the objects. Consistent with previous object mSRE studies that included remember/know judgments in the memory test (Turk et al., 2013; van den Bos et al., 2010) provide further insight here. These studies reported that the majority of ‘recognise’ responses for both self- and other-owned objects were ‘remember’ as opposed to ‘know’ responses (Turk et al., 2013; van den Bos et al., 2010). ‘Remember’ responses are trials on which participants reported that they both recognised the object *and* experienced a conscious, episodic recollection of the object from the encoding task, whereas ‘know’ responses are trials for which participants report a sense of knowing without any specific recollective experience. These recollective experiences may involve information about the ownership cue that appeared with the object and the participant’s response to that cue, and participants could alter their recognition criterion based on this ownership-relevant information from recollective experiences. Relative to a full-attention control group, Turk et al. (2013) reported that participants whose attention was divided during the encoding task showed no object mSRE in CHR and a substantially lower rate of ‘remember’ responses. It is possible that less ownership-related information was encoded and later recollected in the divided attention condition of Turk et al. (2013), with participants applying ownership-related decisional biases on fewer trials during the memory test. Future work could apply a similar divided attention manipulation to test whether recollective experience contributes to the decisional biases observed in our study.

Criterion differences are thought to reflect relatively flexible decision-making processes that are sensitive to task demands, environmental context, and participant characteristics (Schurgin, 2018; Kantner & Lindsay, 2010, 2012). In the object mSRE paradigm, the presence or absence of ownership-related decisions in the memory test may be particularly important. In most previous studies using the Cunningham et al. (2008) ‘shopping task’, participants were asked to indicate whether or not they recognised each object, but *not* prompted to identify each object’s owner during the memory test. In those conditions, it is possible that participants were more motivated to recognise their own compared with others’ objects. Therefore, they may have adopted a more liberal recognition criterion for objects they associated with the self compared with the other. Such a bias would inflate hit rates for self-owned objects relative to other-owned objects, producing the typical object mSRE pattern in CHR measures based on a shared false alarm rate. Similarly, when the memory test has a remember/know component, participants may adopt a more liberal threshold for responding ‘remember’ for self-owned objects than for other-owned objects, resulting in a higher frequency of ‘remember’ hits for self-owned objects compared with other-owned objects (as reported in (Turk et al., 2013; van den Bos et al., 2010).

By contrast, participants in our study knew that each time they responded ‘recognise’ to an object, they would be prompted to identify the object’s owner as either themselves or the other (with no ‘unsure’ response option). This fundamentally changes the nature of the decision and may have affected the perceived social dynamics of the task, impacting on the source memory scores. One possibility is that participants believed it would be socially undesirable to claim ownership mistakenly over the other’s objects, and altered their decision-making strategy accordingly. Adopting a more conservative recognition criterion for self- compared with other-owned objects reduces the number of trials on which this mistake is possible. Likewise, a bias toward attributing ownership of genuinely ‘old’ objects to the other-referent rather than the self reduces the likelihood of mistakenly ‘claiming’ the other’s objects. These biases would also tend to *lower* the hit rate for self-owned objects relative to other-owned objects, consistent with our source-specific hit rate data showing a reversal of the typical object mSRE. As such, our inclusion of an ownership-judgment component in the memory test could explain why we did not replicate the typical finding of a higher CHR for self- compared with other-owned objects. Future studies could test this account by experimentally manipulating the social visibility of participants’ ownership-identification responses in the memory test. For example, this pattern of biases should be stronger when participants believe their responses are visible to the other-referent than when they believe their responses are fully anonymous.

Our findings are similar to those of a recent study using an object-classification paradigm (Golubickis et al., 2018; Vella et al., in preparation). Golubickis et al. (2018) informed participants that one category of objects (pens or pencils) belonged to them and the second belonged to the other person. Participants were presented with a sequence of exemplars from the two categories and instructed to classify each one as self-owned or other-owned as quickly and accurately as possible. Participants were significantly faster to classify self- compared with other-owned objects. Golubickis et al. (2018) applied hierarchical drift diffusion modelling (Wiecki et al., 2013) to separate perceptual processing speed from response bias, and reported that response bias best accounted for the self-owned advantage in response time. Although their study is consistent with ours regarding an effect of ownership on decisional bias, their participants were biased in favour of ascribing self-ownership whereas ours were biased in favour of ascribing other-ownership. The object allocation task in Golubickis et al. (2018) was relatively easy and participants achieved near-ceiling accuracy, which may have rendered redundant any social desirability motivations regarding ownership judgements. Hierarchical drift diffusion modelling as an approach to decisions in memory retrieval could offer further nuanced insights such as the adjustment of drift rates in the rate of evidence accumulation to reach a decision threshold in relation to the criterion (for example, White & Poldrack, 2014). Moving towards computational modelling techniques, it would be interesting to test whether the response bias pattern reverses in more difficult versions of this task where errors in retrieval may be expected. For example, when using degraded images of objects at either the allocation or the retrieval stage, hierarchical linear ballistic model outcomes indicated that a caution decision threshold for self- compared with other-owned objects potentially accounts for self-relevance memory advantages (Vella et al., in preparation). This suggests decision-level factors contribute to the mSRE and potentially play a larger role than early attentional factors.

### Null results for Recognition Sensitivity

Previous work suggests the object mSRE arises from enhanced attentional processing, manifesting as enhanced recognition, of self- compared with other-owned objects during the encoding task (Turk et al., 2013; Turk et al., 2011a, b). In the context of previous literature, enhanced attentional processing should strengthen item memory, thus facilitating recall (Hirshman, 1995; Yonelinas et al., 1992). Accordingly, ownership should impact recognition sensitivity, thought to represent the strength of the memory match signal for genuinely old relative to new objects, independent of any decisional bias (Lockhart & Murdock, 1970; Shapiro, 2014). Although our null findings for recognition sensitivity challenge this explanation of the object mSRE, they should be interpreted cautiously. Statistical power was relatively high in the current study, but a Type II error is still possible, particularly if the true effect size is in fact small. Overall, a strong conclusion that self-ownership provides no recognition sensitivity advantage relative to other-ownership would require replication, ideally with large sample sizes and conducted across different laboratory contexts.

### Identity of other-referent modulates Decisional Bias

We found the identity of the other modulated the effect of ownership on recognition criterion. Participants showed a more conservative recognition criterion for self- compared with other-owned objects, but this criterion difference was significantly larger when the other-referent was the participant’s mother compared with a stranger. In a previous study using only CHR data, we found no effect of other type for a majority-female Western-background sample but a strong effect of other type for Asian-background participants (Sparks et al., 2016a). It is possible that Western-background participants’ decisional biases are sensitive to the identity of the other, consistent with individual differences in criterion for word memory reported by Kantner and Lindsay (2012), but this effect is not readily detectable in CHRs alone. Indeed, CHRs in the current study showed no other-type effects. The social desirability account of recognition criterion differences described above could explain the stronger ownership-related decisional bias in the mother condition. Participants may have believed that mistakenly claiming ownership of their own mother’s possessions would appear *especially* socially undesirable. Alternatively, given that our participants reported high levels of closeness to their mothers, they may have been motivated to ascribe ownership of objects to the mother (by responding ‘recognise’ and then ‘Mother’s’) rather than ‘discarding’ the objects (by responding ‘do not recognise,’ which implies nobody owns the object at hand).

Consistent with this, self-reported ratings of Closeness and Similarity to mother were positively associated with shifts in decisional bias (criterion) for self- versus other owned objects. For the stranger condition, ratings of Closeness and Similarity were low despite the provision of information, and there was no such association with shifts in decisional bias. Although further work is needed to clarify, this could indicate that information about the other does not translate to a perception of closeness or similarity, though it could be responsible for the abolition of the object mSRE in terms of Recognition and Source memory (Clarkson et al., 2022; Sparks et al., 2016a).

We suggest that the identity of the other, and more specifically the relationship between the self and the other in these tasks, is an important avenue for future work. Clarkson et al. (2022) showed that the prominence of the other is an important modulator of the mSRE, even when the other’s presence is virtual. Moreover, investigating the shopping task as a function of age, Clarkson et al. (2024) report that older participants are less likely to demonstrate the mSRE, suggesting a developmental trajectory in self-reference effects. Sui et al. (2007; 2013, 2014) found gradations in self-prioritisation effect using a perceptual matching task, depending on closeness of relationship to the other (self, mother/friend, stranger), so it is possible the same variations apply for the mSRE, depending on closeness, the nature of the relationship, and the changing nature of self-reference across the lifespan. More generally, it may be said that self-reference effects fit within the broader scope of findings indicating that the presence of others modulates our behaviour and cognition functions, including action observation (Bertenthal et al., 2006; Sparks et al., 2016b), joint action (Sebanz et al., 2006) and gaze attention cueing effects (Bayliss et al., 2005; Frischen et al., 2007).

### Generalisability

We opted for female gender across all persons in the task for several reasons. Female participants show larger action observation (Cheng et al., 2008), empathic pain ( Yang et al., 2009) and gaze cuing (Alwall et al., 2010; Bayliss et al., 2005; Frischen et al., 2007) effects, suggesting at a group level they may be more sensitive to social factors within the environment. The majority of previous object mSRE studies tested majority-female samples (Cunningham et al., 2008, 2011; Englert & Wentura, 2016, Experiments 1, 3–6; Grisdale et al., 2014, Experiment 1; Kim & Johnson, 2012; Sparks et al., 2016a; Turk et al., 2013; van den Bos et al., 2010); gender-balanced samples (Englert & Wentura, 2016, Experiment 2; Kim & Johnson, 2014) and majority-male samples (Grisdale et al., 2014, Experiment 2) are relatively rare. Finally, Yue et al. (2020) showed that in trait recognition response times of female participants are faster than those of males. By recruiting only female participants we controlled for gender of participant within the study, as well as matched the gender of the close other (mother), the stranger, and the participant. Given these considerations, we were confident that the mother would represent an effective ‘close other’ for our participants, and our self-reported closeness and similarity ratings support this. The choice, however, does limit the generalisability of our findings. Future studies can confirm whether the same pattern of results occurs for male participants and when the other-referent’s gender differs from the participant’s.

Second, our sample consisted of Caucasian participants with a Western cultural background recruited at an Australian university. The aim of this recruitment strategy was to facilitate straightforward comparison with the majority of previous work on the object mSRE that has been conducted in Western countries (Cunningham et al., 2008, 2011; Englert & Wentura, 2016; Grisdale et al., 2014; Kim & Johnson, 2012, 2014; Turk et al., 2013; van den Bos et al., 2010). Sparks et al. (2016a), however, showed that Western participants demonstrated higher recognition accuracy for self- than other-referenced objects when the other was a stranger or their mother. Participants from Asian (Chinese, Indonesian, and Malaysian) background showed a reversal of this pattern when the other was their mother. This was attributed to possible cultural differences in self-construals, with Asian cultures postulated to have closer associations with their group members (inter-dependence) than participants from Western cultures. This is consistent with the findings of Feng et al. (2013), based on Chinese participants, that mPFC activation to mother-owned objects was as strong as activation to self-owned objects.

It remains an open question whether our findings will generalise to participants with other cultural backgrounds. We have reported that Western-background and Asian-background participants show different patterns memory performance in the Cunningham et al. (2008) object mSRE paradigm (Sparks et al., 2016a), and numerous studies show Western-versus-Asian cultural differences in other SRE paradigms (Ng et al., 2010; Ng & Lai, 2009; Sui et al., 2007; Zhu et al., 2007). Current models in cultural cognition and neuroscience emphasise the interaction between cultural differences and the particular context of the task (Han et al., 2013; Kitayama & Uskul, 2011; Markus & Kitayama, 2010). Of particular note are cultural priming studies with Western/Asian bicultural participants, which suggest that when verbal and/or visual cues of one culture are salient, self-related biases in task performance tend to shift toward the ‘primed’ culture (Huff et al., 2013; Ng et al., 2010; Sui et al., 2007). This shows that the way that self-association effects manifest behaviourally is flexible within an individual, and seems consistent with the flexible nature of decisional biases. We suggest that separating sensitivity and criterion will be fruitful in future studies investigating the mechanism(s) of cultural differences in the OS object mSRE RE.

It is also possible that the specific decisional biases we observed reflect Australian culture. In common with the USA and the UK where samples for most previous studies were recruited, Australian culture values independence and autonomy, but departs in its relatively strong emphasis on egalitarianism (Feather, 1989; Shavitt et al., 2006; Triandis, 1995). For example, there is a particularly strong tendency in Australian culture to censure individuals who present themselves as better than, more accomplished than, or wealthier than others, colloquially labelled ‘tall poppy syndrome’ (Feather, 1989, 1994; Peeters, 2004). This may contribute to our participants’ bias away from responding ‘mine’ in the memory test.

## Conclusion

In summary, we found evidence that object ownership modulates recognition bias, but not recognition sensitivity, using the Cunningham et al. (2008) ‘shopping task’ with a sample of Western, female participants. Participants adopted a more conservative criterion for distinguishing self-owned objects from foils (new, unowned objects) compared with discriminating other-owned objects from foils. These findings suggest that the ‘typical’ ownership self-reference effect (higher hit rates for self-owned compared with other-owned objects) could arise via ownership-based changes in decisional processing during the recognition memory test.

## Data Availability

The data and materials for all experiments are available from the authos. The experiment was not preregistered.
